# Progress achieved in restricting the marketing of high-fat, sugary and salty food and beverage products to children

**DOI:** 10.2471/BLT.15.158667

**Published:** 2016-04-27

**Authors:** Vivica I Kraak, Stefanie Vandevijvere, Gary Sacks, Hannah Brinsden, Corinna Hawkes, Simón Barquera, Tim Lobstein, Boyd A Swinburn

**Affiliations:** a Department of Human Nutrition, Foods, and Exercise, Virginia Tech, 223 Wallace Hall, 295 West Campus Drive, Blacksburg, Virginia 24061, United States of America.; b School of Population Health, University of Auckland, Auckland, New Zealand.; c WHO Collaborating Centre for Obesity Prevention, Deakin University, Burwood, Australia.; d World Obesity Federation, London, England.; e Centre for Food Policy, City University London, London, England.; f National Institute of Public Health of México, Cuernavaca, Mexico.

## Abstract

In May 2010, 192 Member States endorsed Resolution WHA63.14 to restrict the marketing of food and non-alcoholic beverage products high in saturated fats, trans fatty acids, free sugars and/or salt to children and adolescents globally. We examined the actions taken between 2010 and early 2016 – by civil society groups, the World Health Organization (WHO) and its regional offices, other United Nations (UN) organizations, philanthropic institutions and transnational industries – to help decrease the prevalence of obesity and diet-related noncommunicable diseases among young people. By providing relevant technical and policy guidance and tools to Member States, WHO and other UN organizations have helped protect young people from the marketing of branded food and beverage products that are high in fat, sugar and/or salt. The progress achieved by the other actors we investigated appears variable and generally less robust. We suggest that the progress being made towards the full implementation of Resolution WHA63.14 would be accelerated by further restrictions on the marketing of unhealthy food and beverage products and by investing in the promotion of nutrient-dense products. This should help young people meet government-recommended dietary targets. Any effective strategies and actions should align with the goal of WHO to reduce premature mortality from noncommunicable diseases by 25% by 2025 and the aim of the UN to ensure healthy lives for all by 2030.

## Résumé

En mai 2010, 192 États membres ont ratifié la Résolution WHA63.14 pour restreindre, à l'échelle internationale, la commercialisation des produits alimentaires et boissons non alcoolisées riches en graisses saturées, en acides gras *trans*, en sucres libres et/ou en sel, destinés aux enfants et aux adolescents. Nous avons étudié des initiatives d'organisations de la société civile, de l'Organisation mondiale de la Santé (OMS) et de ses bureaux régionaux, d'autres agences de l'Organisation des Nations unies (ONU), d'institutions philanthropiques et de groupes industriels internationaux, menées entre 2010 et début 2016 en vue d’aider à réduire la prévalence de l'obésité et des maladies non transmissibles liées à l'alimentation chez les jeunes. En fournissant aux États membres des outils et des conseils utiles en matière technique et pour l'adoption de mesures politiques, l'OMS et d'autres agences de l'ONU ont contribué à protéger les jeunes face à la commercialisation d'aliments et de boissons de marques, riches en graisses, en sucre et/ou en sel. Les résultats obtenus grâce aux initiatives des autres acteurs étudiés semblent variables et généralement moins solides. Nous suggérons d'accélérer les progrès accomplis vers la mise en œuvre complète de la Résolution WHA63.14, en restreignant davantage la commercialisation des aliments et boissons mauvais pour la santé et en investissant dans la promotion de produits denses sur le plan nutritionnel. Cela permettrait d'aider les jeunes à atteindre les objectifs nutritionnels recommandés par les gouvernements. Pour être efficaces, les stratégies et mesures adoptées doivent être cohérentes avec l'objectif de l'OMS visant à réduire la mortalité prématurée due aux maladies non transmissibles de 25% d'ici 2025 et avec l'objectif de l'ONU visant à permettre à tous de vivre en bonne santé d'ici 2030.

## Resumen

En mayo de 2010, 192 Estados Miembros aprobaron la Resolución WHA63.14 para limitar la promoción de alimentos y bebidas no alcohólicas con elevadas cantidades de grasas saturadas, ácidos grasos trans, azúcares libres y/o sal dirigida a los niños y adolescentes de todo el mundo. Se examinaron las medidas tomadas entre 2010 y principios de 2016 por parte de grupos de la sociedad civil, la Organización Mundial de la Salud (OMS) y sus sedes regionales, otras organizaciones de las Naciones Unidas, instituciones filantrópicas e industrias transnacionales para contribuir a la reducción de la prevalencia de la obesidad y enfermedades no contagiosas relacionadas con la alimentación entre los jóvenes. Mediante las directrices y herramientas políticas y técnicas correspondientes ofrecidas a los Estados Miembros, la OMS y otras organizaciones de las Naciones Unidas han ayudado a proteger a los jóvenes de la promoción de productos alimentarios y bebidas de marca con elevadas cantidades de grasa, azúcar y/o sal. Los progresos realizados por los otros participantes investigados parecen ser desiguales y, en general, menos sólidos. Nuestra sugerencia es que el progreso hacia la implementación completa de la Resolución WHA63.14 se acelere limitando aún más la promoción de productos alimentarios y bebidas insanos y se invierta en la promoción de productos altamente nutritivos. Esto debería ayudar a los jóvenes a alcanzar los objetivos nutricionales recomendados por los gobiernos. Todas las estrategias y medidas eficaces deberían ajustarse a la meta de la OMS de reducir la mortalidad prematura provocada por enfermedades no contagiosas en un 25% en 2015 y el objetivo de las Naciones Unidas de garantizar una vida sana para todos en 2030.

## ملخص

شهد شهر مايو/أيار من عام 2010 تأييد القرار رقم WHA63.14 من جانب 192 دولة من الدول الأعضاء، والذي يقضي بفرض القيود على تسويق المنتجات الغذائية والمشروبات غير الكحولية التي تحتوي على نسب عالية من الدهون المشبعة، والدهون المشبعة المتحولة، والسكريات الحرة و/أو الملح للأطفال والمراهقين على مستوى العالم. ولقد بحثنا في الإجراءات التي اتخذت بين عامي 2010 وأوائل عام 2016 – من قبل منظمات المجتمع المدني، ومنظمة الصحة العالمية ومكاتبها الإقليمية، والمنظمات الأخرى التابعة للأمم المتحدة، والمؤسسات الخيرية، والصناعات عبر الوطنية – للمساعدة في التخفيف من معدلات انتشار البدانة والأمراض غير المعدية ذات الصلة بالنظام الغذائي في أوساط الصغار. وقد ساعدت منظمة الصحة العالمية والمنظمات الأخرى التابعة للأمم المتحدة في حماية الشباب من مخاطر تسويق المنتجات الغذائية والمشروبات ذات العلامات التجارية والتي تحتوي على نسب عالية من الدهون والسكر و/أو الملح، من خلال تقديم توجيهات وأدوات فنية تتعلق بالسياسات للدول الأعضاء. أما التقدم الذي أحرزته الأطراف الفاعلة الأخرى التي تحرينا عنها فيبدو متباينًا وأقل فعالية بشكل عام.

وفي هذا الصدد، فإننا نقترح الإسراع من وتيرة التقدم المحقق باتجاه التنفيذ الكامل للقرار رقم WHA63.14 عن طريق فرض المزيد من القيود على تسويق المنتجات الغذائية والمشروبات غير الصحية، وعن طريق الاستثمار في الترويج لمنتجات غنية بالمواد المغذية.

هذا الأمر من شأنه مساعدة الصغار على تحقيق الأهداف الغذائية الموصى بها من الحكومة. وينبغي على أي استراتيجيات وإجراءات فعالة أن تكون متوازية مع هدف منظمة الصحة العالمية للحد من الوفيات المبكرة بسبب الأمراض غير المعدية بنسبة 25‏% بحلول عام 2025 وهدف الأمم المتحدة لضمان تمتع جميع البشر بحياة صحية بحلول عام 2030.

## 摘要

2010 年 5 月，192 个会员国通过了 WHA63.14 决议，在全球范围内限制向儿童和青少年推销含有大量饱和脂肪、反式脂肪酸、游离糖和/或盐的食品和非酒精饮料。 我们对 2010 年至 2016 年初，民间社会组织、世界卫生组织 (WHO) 及其地区办事处、其他联合国 (UN) 机构、慈善机构和跨国性行业所采取的相关措施进行了审查。这些措施旨在降低年轻人群的肥胖症以及与膳食相关的非传染性疾病的发生率。 通过向会员国提供相关技术、政策指导以及工具，世界卫生组织 (WHO) 和其他联合国 (UN) 机构协助会员国限制向年轻人群推销高脂肪、高糖和/或高盐的品牌食品和饮料产品。 通过调查我们发现，其他活动方取得的进展变数较大，且通常不够强劲。 我们建议可通过进一步限制不健康食品和饮料的推销以及加大对高营养产品的推广，加快推进全面贯彻 WHA63.14 决议所取得的进展。 这将有助于年轻人群达到政府推荐的膳食目标。 任何有效策略和措施均应该以世界卫生组织 (WHO) 的目标为准——到 2025 年，将由非传染性疾病导致的过早死亡率降低 25%，同时应符合联合国 (UN) 的到 2030 年确保所有人过上健康生活的目标。

## Резюме

В мае 2010 года 192 государства-участника поддержали Резолюцию WHA63.14, ограничивающую сбыт пищевых продуктов и безалкогольных напитков с высоким содержанием насыщенных жиров, трансжирных кислот, свободных сахаров и/или соли детям и подросткам во всем мире. Авторы проанализировали мероприятия, проведенные в период между 2010 годом и началом 2016 года группами гражданского общества, Всемирной организацией здравоохранения (ВОЗ) и ее региональными отделениями, другими организациями ООН, благотворительными учреждениями и транснациональными предприятиями. Эти мероприятия были нацелены на снижение распространенности ожирения и неинфекционных заболеваний, связанных с нарушением питания, среди молодых людей. Предоставив необходимые технические и стратегические руководящие указания и инструменты государствам-участникам, ВОЗ и другие организации ООН совершили вклад в защищенность молодых людей от сбыта фирменных пищевых продуктов и напитков с высоким содержанием жиров, сахара и/или соли. В результате исследования было обнаружено, что другие действующие субъекты достигли переменного и в целом менее устойчивого успеха. Авторы предполагают, что ускорить прогресс в плане выполнения Резолюции WHA63.14 во всех ее аспектах позволило бы дальнейшее ограничение сбыта нездоровых продуктов питания и напитков и инвестирование в популяризацию продуктов с высоким содержанием питательных веществ. Благодаря этому молодые люди смогут достичь целей в области питания населения, рекомендованных государством. Все эффективные стратегии и меры должны быть согласованы с целью ВОЗ снизить преждевременную смертность от неинфекционных заболеваний на 25% к 2025 году и целью ООН обеспечить здоровый образ жизни для всех к 2030 году.

## Introduction

In May 2010, the 192 Member States of the World Health Organization endorsed Resolution WHA63.14. The aim of this resolution is to restrict the marketing of unhealthy food and non-alcoholic beverage products to children and adolescents to reduce the prevalences of overweight, obesity and diet-related noncommunicable diseases.^1^ Globally, about 42 million children younger than 5 years and 155–200 million school-aged children are overweight or obese.^2^^,^^3^ Nearly 2.7 billion adults will be overweight or obese by 2025.^4^ The rapid increase in the prevalence of overweight and obesity among children in low- and middle-income countries has been described as a time bomb that could cause immense damage to health-care systems worldwide.^5^

We examined the actions taken, between 2010 and early 2016, by the World Health Organization (WHO) – via its headquarters and six regional offices – and other United Nations (UN) organizations to offer technical and policy guidance to Member States to implement Resolution WHA63.14. We subsequently assessed the extent of the same resolution’s implementation by the national governments of Member States and investigated the supportive actions of relevant civil society organizations, philanthropic institutions and transnational industrial actors – e.g. food and beverage manufacturers, retailers, restaurant companies and industrial trade groups. We focused on actions designed to restrict young people’s exposure to the powerful and pervasive marketing of branded unhealthy food and non-alcoholic beverage products – i.e. products that are high in saturated fats, trans fatty acids, free sugars and/or salt. Below, we present the results of these investigations and suggest strategies and actions to accelerate the implementation of Resolution WHA63.14.

## Marketing, diet and health risks

A robust evidence base accumulated between 2003 and 2013 demonstrated how the extensive and persistent exposure to the powerful marketing of unhealthy food and beverage products could affect the food and drink preferences and purchase requests of children and adolescents.^6^^–^^10^ Rigorous reviews have documented how the often sophisticated and integrated marketing communications of transnational food and drink industries continue to influence the dietary behaviours of young people and contribute to energy-dense and nutrient-poor diets, increased risks of unhealthy weight gain and negative health outcomes.^7^^–^^10^

As they have a biological preference for sweet and salty tastes, infants and young children younger than 5 years are considered especially vulnerable to marketing practices that promote sugary and salty food and beverage products.^11^ Children’s recognition of branded food logos increases with age^12^ and overweight children are more likely to recognize the brands of fast food restaurants than those of other food and beverage products.^13^ Compared with other children, those who recall branded unhealthy food and beverage products have stronger preferences for such products.^14^ Children’s knowledge of unhealthy food and beverage products increases their obesity risk.^15^ Adolescents aged 12–18 years have more discretionary income than children and are uniquely susceptible to an immersive and evolving digital marketing landscape that normalizes unhealthy food and beverage products.^16^ Such marketing is also associated with materialistic values and aspirational lifestyles that often have harmful impacts among young people.^17^

## Technical and policy guidance

**Fig. 1:**
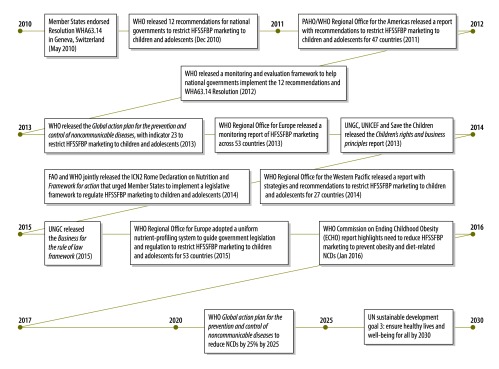
Support to restrict the marketing of branded high-fat, salty and/or sugary food and beverage products to children and adolescents, 2010–2016

FAO: Food and Agriculture Organization of the United Nations; HFSSFBP: high-fat, salty and/or sugary food and beverage products; ICN2: Second International Conference on Nutrition; INFORMAS: International Network for Food and Obesity/Non-communicable Diseases Research, Monitoring and Action Support; NCDs: noncommunicable diseases; PAHO; Pan American Health Organization; UN: United Nations; UNGC: United Nations Global Compact; UNICEF: United Nations Children’s Fund; WHA: World Health Assembly; WHO: World Health Organization.

Note: The support shown was provided by the World Health Organization and other United Nations agencies to Member States. The figure also shows some goals to be achieved by 2030.

Following the adoption of Resolution WHA63.14,^1^ WHO released 12 recommendations^18^ that encouraged national governments to: (i) implement policies to restrict the marketing of unhealthy food and beverage products in settings where children spend time; (ii) reduce the impact of the cross-border marketing of such products; and (iii) monitor the nature and extent of the marketing of such products and the effectiveness of government regulations to restrict young peoples’ exposure to – and the harmful impacts of – such marketing. We summarize below the evidence of the leadership provided, between 2010 and early 2016, by WHO’s headquarters and regional offices and other UN agencies – via the provision of technical and policy guidance and tools to assist Member States in the protection of young people from the marketing of unhealthy food and beverage products (Fig. 1).

In 2011, the Pan American Health Organization (PAHO) adapted WHO’s 12 recommendations for use in 47 countries.^19^ In 2012, WHO developed a comprehensive framework that offered Member States the technical support they might need to restrict and monitor marketing practices that promoted unhealthy branded food and beverage products across media platforms, various settings and country borders.^20^ In 2013, however, WHO’s Regional Office for Europe released data, from monitoring activities in 53 countries, that demonstrated many weaknesses in the framework – e.g. an overreliance on voluntary pledges, the exploitation of loopholes by food, beverage and restaurant industries and insufficient government regulation and enforcement.^21^

In 2012, inspired by the protect, respect and remedy accountability framework previously developed by the UN, the United Nations Children’s Fund (UNICEF), the UN Global Compact and Save the Children jointly released model business practices to protect children’s rights.^22^ These practices were reinforced in 2015 by the UN Global Compact’s release of the *Business for the rule of law* framework.^23^

In 2013, WHO released the *Global action plan for the prevention and control of noncommunicable diseases, 2013–2020*. This plan included an indicator to monitor the restriction of the marketing of unhealthy food and beverage products to individuals aged 0–18 years, to help reduce premature mortality from noncommunicable diseases by 25% by 2025.^24^ WHO asked policy-makers and decision-makers within national governments to mobilize the political will, allocate the financial resources and create the transparent accountability mechanisms needed to ensure national legislation to monitor this marketing indicator.^24^^,^^25^ In 2014, WHO’s Regional Office for the Western Pacific outlined strategies that national governments could use to regulate practices that encouraged the marketing of unhealthy food and beverage products and counteract the attempts of some food industries to undermine the development of supportive policies.^26^

Over 2200 people, including representatives of 170 Member States, attended the Second International Conference on Nutrition in November 2014. The attendees endorsed the Rome Declaration on Nutrition that called for “improvements in diet and nutrition and requiring a relevant legislative framework … while avoiding inappropriate marketing and publicity of foods and non-alcoholic beverages to children, as recommended by Resolution WHA63.14”.^27^ The conference’s *Framework for action* urged national governments to implement coherent policies and coordinated actions across sectors to “regulate the marketing of food and non-alcoholic beverages to children according to the WHO’s 2010 recommendations.”^28^

In 2015, WHO’s Regional Office for Europe released a food- and nutrient-based profiling model to help governments reduce the marketing of unhealthy food and beverage products to young people.^29^ This model is currently being adapted for use by the Regional Offices for the Eastern Mediterranean and Western Pacific (Chizuru Nishida, WHO, personal communication, 2015). In 2016, PAHO released a nutrient-profile model developed for several purposes, including the reduction of the marketing of unhealthy food and beverage products to young people.^30^

In 2016, WHO’s *Report of the commission on ending childhood obesity*^31^ recognized that the “settings where children and adolescents gather and the screen-based offerings they watch, should be free of marketing of unhealthy foods and sugar-sweetened beverages”. The report also noted, with concern, that Member States had failed to give significant attention to Resolution WHA63.14 and requested that they address this issue. The Commission urged Member States to: (i) implement the recommendations to restrict young people’s exposure to – and the power of – the marketing of unhealthy food and beverage products; (ii) cooperate to reduce the impact of the cross-border marketing of such products; and (iii) develop nutrient-profiling systems to identify and regulate such products.^31^

## Progress

### National governments

Since 2010, many national governments have favoured self-regulation by industry to restrict the marketing of unhealthy food and beverage products.^32^^–^^34^ A survey of 59 countries in 2011 showed that, although national governments had developed or implemented some relevant statutory measures, the nature and extent of the marketing restrictions differed across countries and regions.^35^ No Member State has implemented comprehensive legislation or enforced mandatory regulations to prohibit the marketing of fatty, salty and/or sugary branded food and non-alcoholic beverage products to young people.

### Transnational industrial actors

**Fig. 2:**
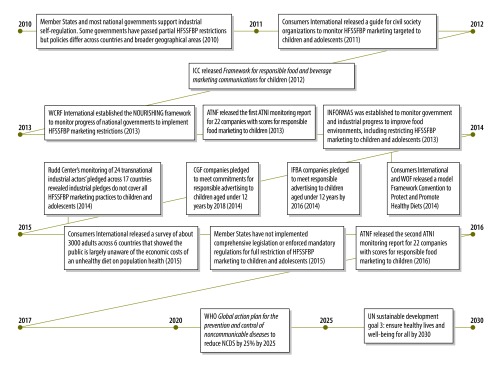
Progress achieved in restricting the marketing of branded high-fat, salty and/or sugary food and beverage products to children and adolescents, 2010–2016

ATNF: Access to Nutrition Foundation; ATNI: Access to Nutrition Index; CGF: Consumer Goods Forum; HFSSFBP: high-fat, salty and/or sugary food and beverage products; ICC: International Chamber of Commerce; IFBA: International Food and Beverage Alliance; INFORMAS: International Network for Food and Obesity/Non-communicable Diseases Research, Monitoring and Action Support; NCDs: noncommunicable diseases; UN: United Nations; WCRF: World Cancer Research Fund; WHO: World Health Organization; WOF: World Obesity Federation.

Note: The progress shown was achieved by civil society organizations, national governments in Member States and transnational industrial actors. The figure also shows some goals to be achieved by 2030.

In 2012, the International Chamber of Commerce released a *Framework for responsible food and beverage marketing communications* that reflected principles of a broader International Code on Advertising. These principles, which are supported by the World Federation of Advertising and other International Chamber of Commerce members, were designed to promote ethical and legal standards for voluntary self-regulation of business marketing practices (Fig. 2).^36^ Many of the largest food and beverage manufacturers worldwide have pledged to restrict the advertising – but not all forms of the marketing – of branded food and beverage products with high levels of fat, sugar and/or salt to children younger than 12 years. In 2014, 11 companies within the International Food and Beverage Alliance reported that they had stronger voluntary pledges than in 2011,^37^ continued to support third-party compliance monitoring – by Accenture (Dublin, Ireland) – and promised that, by 2016, they would only advertise products to children younger than 12 years that met common nutrition criteria based on scientific dietary guidelines.^37^ In 2014, the members of the Consumer Goods Forum – a consortium of over 400 food manufacturers and retailers across 70 countries – pledged that, by 2018, they would stop advertising, to children younger than 12 years, food and beverage products that do not meet science-based nutrition criteria (Fig. 2).^38^

The Access to Nutrition Foundation – a private health-related philanthropic institution –released its first *Access to nutrition index*, a global monitoring report, in 2013.^39^ This report rated 22 transnational food and beverage manufacturers across nine indicators, one of which was responsible food and beverage marketing practices. A score of 10 represented the highest level of coordinated actions to support responsible marketing to children and adults. Among the companies within the International Food and Beverage Alliance, the scores for responsible marketing fell from 5.2 for Danone, to 4.8 for Unilever, 4.6 for PepsiCo, 4.4 for Kraft Foods, 4.1 for The Coca-Cola Co., 4.0 for Nestlé and 2.7 for Kellogg. In the corresponding report for 2016, many companies’ marketing scores were higher than their 2013 values, with Danone (8.5), Unilever (7.7) and Nestlé (7.4) leading the marketing index.^40^ Compared with the 2013 report, the 2016 report indicated no measurable improvement in the prioritization and implementation of a global policy for the responsible marketing of healthy food and beverage products. Both the 2013 and 2016 reports of the Access to Nutrition Foundation recommended that transnational food and beverage firms increase their transparency and accountability to enable independent monitoring bodies to assess their progress and develop corrective actions.

No company has yet restricted the marketing of unhealthy food and beverage products comprehensively – i.e. in all settings, for all practices and across all media platforms – either to children younger than 12 years or to adolescents aged 12–18 years. Moreover, food retailers, entertainment companies and many firms that operate fast food restaurants in chain franchises have failed to adopt any global commitments similar to the pledges made by the Consumer Goods Forum, the International Food and Beverage Alliance or the International Chamber of Commerce.

Some transnational companies use private-sector alliances to persuade legislators and the public to oppose health-related restrictions on food and drink marketing. These attempts have fuelled government inaction and weakened civil society’s response to the implementation of Resolution WHA63.14.^41^ However, industry actors are partnering with some UN agencies, including UNICEF and the World Food Programme, to address the problems of hunger and undernutrition in countries with rising prevalences of obesity and diet-related noncommunicable diseases.^41^ These partnerships must be carefully vetted to prevent, manage and mitigate any potential conflicts of interest and avoid the risks and consequences associated with the promotion of unhealthy food and beverage products while addressing undernutrition.

### Civil society and philanthropic organizations

The focus of civil society efforts has been to monitor the policies and actions of transnational industrial actors and national governments (Fig. 2). In 2011, Consumers International released a guide to encourage civil society organizations to monitor the marketing of unhealthy food and beverage products that targeted children and adolescents.^42^ Between 2010 and 2015, comprehensive monitoring and evaluation reports published by academics and civil society organizations^32^^,^^43^^–^^46^ showed several inconsistencies among the voluntary pledges used by various transnational industrial actors across geographical areas. These reports emphasized the inherent weaknesses of industry-funded self-regulatory programmes, and decision-makers who lack the authority and resources to hold underperforming or non-compliant businesses accountable for marketing practices that contribute to obesity and diet-related noncommunicable diseases. The Rudd Center’s monitoring of the voluntary pledges of transnational industrial actors across 17 countries and four global areas^46^ revealed that none of the 24 companies investigated had extended pledges to cover all food and/or beverage marketing practices that targeted adolescents aged 14–18 years.

In 2013, the World Cancer Research Fund International established the NOURISHING framework to monitor the progress of national governments to implement restrictions on the marketing of unhealthy food and beverage products.^34^ The International Network for Food and Obesity/Non-communicable Diseases Research, Monitoring and Action Support (INFORMAS) was established to monitor the progress of national governments and diverse industrial sectors in improving the healthiness of food – including the restriction of marketing of unhealthy food and beverage products to children and adolescents.^45^^,^^47^ In 2014, Consumers International and the World Obesity Federation outlined guiding principles, general obligations and 14 specific articles for a model Framework Convention to Protect and Promote Healthy Diets^48^ and advocated for WHO to work with Member States to enact mandatory actions and strong accountability structures to address unhealthy diets.

## Priority strategies and actions

Table 1 summarizes our recommendations for policy development, implementation, monitoring and evaluation by diverse stakeholders. If followed, these recommendations should help in the full implementation of Resolution WHA63.14, the reduction of the premature mortality from noncommunicable diseases by 25% by 2025^25^ and the sustainable development goal to achieve healthy lives for all by 2030.^49^

**Table 1:** Priority strategies and actions for accelerating progress towards full implementation of Resolution WHA63.14 by 2025

Actors	Policy development	Policy implementation	Policy monitoring and evaluation
**Health-related civil society and philanthropic organizations**	Provide advocacy and social-movement building skills to create an enabling environment for national governments and UN agencies to uphold strong legislation to support this issue. Offer clear guidelines for voluntary engagement and disengagement with transnational industrial actors through alliances and partnerships.	Use media advocacy to raise public awareness about the global costs of an unhealthy diet, to strengthen public support for restrictions on HFSSFBP and establish strong accountability systems that include financial penalties for non-compliant companies and industry sectors that do not protect young people from the marketing of HFSSFBP.	Conduct and publish independent monitoring and evaluations of progress achieved by Member States and transnational industrial actors to restrict the marketing of HFSSFBP.
**National governments of Member States**	Set clear goals and targets to restrict young people’s exposure to branded HFSSFBP.	Enact legislation and regulation in accordance with Resolution WHA63.14 and the 2016 ECHO report and establish performance targets that use a standardized, government-defined, nutrient-profiling model across national borders and continents, accompanied by a timeline for expected outcomes.	Strengthen voluntary industry self-regulatory programmes, support the monitoring of expenditure on – and practices in – the marketing of HFSSFBP and enable regulatory bodies to hold non-compliant companies accountable for young peoples’ exposure to such products – via all media platforms.
**Transnational industries**	Adopt the UNGC’s Responsible Business Practices and commit to clear goals and targets set by national governments to restrict young people’s exposure to branded HFSSFBP. Protect children and adolescents by not opposing government actions to implement strong legislation and regulation.	Implement competitive business plans to reduce young people’s exposure to branded HFSSFBP, and shift marketing resources and product portfolios from such products towards nutrient-dense products, to help young people meet dietary targets.	Demonstrate transparency and cooperation by sharing relevant information on websites and with independent monitoring bodies to monitor and evaluate progress made to restrict the marketing of HFSSFBP to young people within and across countries and globally.
**WHO headquarters and regional offices**	Support Member States by integrating the marketing of breast-milk substitutes, infant foods and HFSSFBP into a strong Code of Conduct, with long-term funding to support robust monitoring, reporting and accountability systems.	Provide Member States with technical assistance to adopt a standardized, global nutrient-profiling model and to enact policies and legislation to restrict marketing of HFSSFBP to young people.	Publish regular updates on the progress achieved by Member States to fully implement Resolution WHA63.14 by 2025.

ECHO: Commission on Ending Childhood Obesity; HFSSFBP: high-fat, salty and/or sugary food and beverage products; UN: United Nations; UNGC: United Nations Global Compact; WHA: World Health Assembly; WHO: World Health Organization.

### Civil society and philanthropic organizations

Health-related civil society and philanthropic organizations can support national governments in establishing and strengthening regulatory bodies that are empowered to define and restrict the marketing of unhealthy foods and beverages targeted at children and adolescents – and to hold private-sector businesses to account for such marketing. Smaller civil society organizations will need support to develop the effective advocacy and social momentum they will need to build an enabling environment – i.e. one in which national governments and UN agencies may fulfil their commitment to enact and uphold strong legislation. We need clear guidelines for the voluntary engagement of civil society and philanthropic organizations with transnational industrial actors, via alliances and partnerships. We also need such organizations to: (i) use media-based advocacy to raise public awareness about the global costs of an unhealthy diet,^50^ to strengthen public support for restrictions on the marketing of unhealthy food and beverage products; (ii) conduct relevant monitoring and research; and (iii) advocate for strong accountability systems that include financial penalties for non-compliant companies and industrial sectors that fail to protect young people from the marketing of unhealthy food and beverages.^51^

### National governments

Decision-makers in national government should set clear goals to restrict young people’s exposure to unhealthy branded food and beverage products, enact legislation and regulation in accordance with Resolution WHA63.14 and establish performance targets – using a government-defined, standardized nutrient-profiling system that applies across national borders and geographical areas and is accompanied by a defined timeline for expected outcomes. The decision-makers also need to allocate funding to: (i) establish or strengthen voluntary industrial self-regulatory programmes; (ii) support the monitoring of food and beverage marketing expenditures and practices; and (iii) enable regulatory bodies to hold non-compliant companies accountable for young people’s exposure, via all marketing practices and media platforms, to unhealthy food and beverage products.^51^

### Transnational industrial actors

The industrial actors should adopt a comprehensive global policy on responsible marketing to children and adolescents that is accessible to all stakeholders in the public domain. The policy should include all forms of integrated marketing communications – e.g. company-owned, brand-equity mascots and licensed media characters used on in-store food packaging and at points of sale, in-school food marketing, sponsorships, celebrity endorsements, charitable donations and fundraising activities.^18^^,^^20^^,^^47^ Industrial actors should also: (i) commit to clear targets set by national governments to restrict young people’s exposure to unhealthy branded food and beverage products; (ii) use a government-approved nutrient-profiling system to guide the development of acceptable products; (iii) not oppose government actions to implement health-protecting legislation and regulation; (iv) design and implement competitive business plans to shift their marketing resources from unhealthy products to promote profitable but nutrient-dense products – and so help young people meet their recommended dietary targets for fruits and vegetables, whole grains and low-fat or non-fat dairy or dairy-substitute products; and (v) amend existing self-regulatory programmes to protect the diet and health of all individuals aged 18 years and younger. Finally, industrial actors must demonstrate transparency and share relevant information on their websites and with independent monitoring bodies, to facilitate the evaluation of progress to restrict the marketing of unhealthy food and beverage products to young people – within and across countries and globally.

### WHO

WHO should support Member States by integrating the marketing of breast-milk substitutes, infant foods and unhealthy food and beverage products into a strong overarching Code of Conduct for the marketing of healthy foods and beverages – with long-term funding to support robust monitoring, reporting and accountability systems. WHO’s headquarters and its regional offices should provide Member States with technical assistance to adopt a standardized, global food and nutrient-profiling system, enact legislation and policies to restrict the marketing of unhealthy food and beverage products to young people, and publish regular updates on the progress that Member States are making towards the full implementation of Resolution WHA63.14 by 2025.

## Conclusion

The five-year anniversary of Resolution WHA63.14 encourages us to reflect on the progress achieved and further actions required to protect young people from the harmful impacts of the marketing of unhealthy food and beverage products – especially given the rising prevalences of obesity and diet-related noncommunicable diseases worldwide. Several UN agencies offered useful leadership between 2010 and early 2016, by providing technical and policy guidance to Member States. However, no Member State has implemented comprehensive legislation or enforced mandatory regulations to prohibit the marketing of unhealthy food and beverage products to young people. The Access to Nutrition Foundation 2016 scorecard has confirmed that transnational food and beverage firms have not yet implemented a global pledge, to engage in responsible marketing to young people, that covers all practices. Moreover, no industrial sector uses a standardized, government-supported nutrient profiling system to restrict the marketing of unhealthy food and beverage products to those aged 14–18 years. Civil society organizations can develop model pledges to help Member States and industry accelerate progress. National governments and other relevant actors have future opportunities to build policy coherence across settings, sectors and continents and establish healthy food environments for young people. These actions should support the UN Convention on the Rights of the Child, the right to adequate nutrition, the reversal of the current upward trend in the prevalence of undernutrition, obesity and diet-related noncommunicable diseases, and the promotion of healthy lives for all by 2030.
